# Draft Genome Sequences of Staphylococcus sp. Strain CWZ226, of Unknown Origin, and Pseudomonas sp. Strain CVAP#3, Antagonistic to Strain CWZ226

**DOI:** 10.1128/MRA.00688-21

**Published:** 2021-09-16

**Authors:** Brianna L. McCall, J. A. C. Vriezen

**Affiliations:** a Smith College, Department of Biological Sciences, Northampton, Massachusetts, USA; Loyola University Chicago

## Abstract

Many Staphylococcus and Pseudomonas species, such as Staphylococcus aureus and Pseudomonas aeruginosa, are opportunistic human pathogens. However, Pseudomonas species are also known to produce bioactive compounds. Here, we report on the genome sequences of a Pseudomonas isolate and a Staphylococcus species of unknown origin that it inhibits.

## ANNOUNCEMENT

Many non-*aureus* or non-*aeruginosa* Staphylococcus and Pseudomonas species are known emerging pathogens (1–6). However, Pseudomonas strains are also known to produce toxic compounds such as cyclic lipopeptides (7, 8).

A bacterial colony (CVAP#3) was isolated from a soil sample (sampled at 42°19′04.01″N, 72°38′25.60″W, at an altitude of 41.76 m, on 21 November 2014) that had been suspended in 1× phosphate-buffered saline (PBS), plated onto 10% tryptic soy agar (TSA), and incubated for 48 h at 25°C. CVAP#3 inhibited a lawn of Staphylococcus (CWZ226) on TSA at 25°C overnight. CWZ226 is of unknown origin and is maintained in a culture collection at Smith College.

In order (i) to determine the phylogenetic position of strains CVAP#3 and CWZ226 and (ii) to identify loci for bioactive compounds against Staphylococcus, the genomes of both strains were sequenced. To isolate DNA, the Promega Wizard genomic DNA kit was employed (catalog number A1120). The Nextera DNA Flex library preparation kit (catalog number 20018704) was used for library construction. Sequencing was performed at Smith College using the MiSeq reagent kit (cartridge) v3 for 150 cycles (catalog number MS-102-3001) or 600 cycles (catalog number MS-102-3003); this resulted in 5,050,320 and 956,980 raw reads for the paired-end reads of 75-bp and 300-bp sequences, respectively, for the CVAP#3 genome. Similarly, two sequencing runs for the Staphylococcus genome resulted in 2,386,984 and 2,600,230 paired-end reads of 300 bp.

Both sets of paired-end reads were assembled in SPAdes v3.11.1 (9) in careful mode with the -kmer set at 73 (CVAP#3) or default (CWZ226) and a Phred offset of 33. Contigs smaller than 700 bp were removed. Metagenome binning in PATRIC v3.6.9 (10) resulted in 45 contigs and 31 contigs in one bin with 33.1× and 506× coverage for CVAP#3 and CWZ226, respectively. Comprehensive genome analysis and annotation of the CVAP#3 genome in PATRIC resulted in a total genome length of 6,255,897 bp, an *N*_50_ value of 301,990 bp, and an *L*_50_ value of 7. Completeness is 99.1%, the GC content is 58.7%, and a total of 58 tRNAs, 1 rRNA, 1,395 hypothetical proteins, and 4,510 known proteins were found. The analysis of the CWZ226 genome resulted in a total length of 2,557,926 bp, an *N*_50_ value of 209,069 bp, and an *L*_50_ value of 5. Completeness is 99.9%, the GC content is 36.1%, and a total of 57 tRNAs, 6 rRNAs, 520 hypothetical proteins, and 1,854 known proteins were found.

The 16S sequences were used as queries in the NCBI genomic database (11). The highest level of identity was with Pseudomonas fluorescens (99.9%) (12) and Staphylococcus simulans (99.7%) (4) for strains CVAP#3 and CWZ226, respectively. Strict analysis of the Pseudomonas genome with antiSMASH v6.0.0 (13) resulted in seven gene clusters putatively involved in the production of secondary metabolites. Five of these gene clusters are putatively involved in the production of fengycin and two different siderophores, pseudomonine and pseudopyronine, compounds with antimicrobial and anticancer activity. In addition, two of these gene clusters code for bacteriocins and have domains associated with alkaline phosphatases and endonuclease (DUF692 domain).

### Data availability.

This whole-genome shotgun project has been deposited in DDBJ/ENA/GenBank; the accession numbers are JAHMSC000000000 for Pseudomonas sp. strain CVAP#3 and JAHMSB000000000 for Staphylococcus sp. strain CWZ226. All raw sequencing files can be found in the SRA under accession number SRP323427. The strains used (CVAP#3, MC4100, and CWZ226) can be requested by email (jvriezen@smith.edu).
